# Supplementary material for: COVID-19-related stigma among infected people in Sweden; psychometric properties and levels of stigma in two cohorts as measured by a COVID-19 stigma scale
Source: PLoS One. 2023 Jun 21;18(6):e0287341. doi: 10.1371/journal.pone.0287341 (PMC10284413; doi:10.1371/journal.pone.0287341)
Supplement: Attachment — Submitted filename: Response to reviewers.docx [file pone.0287341.s001.docx]

Re Manuscript ID PONE-D-22-35422 titled “Covid-19-related stigma among infected people in Sweden; psychometric properties and levels of stigma in two cohorts as measured by a covid-19 stigma scale”

Dear Dr Nabeel Al-Yateem

Thank you for your feedback and the constructive reviews. We have reacted to all comments and made changes as proposed by the reviewers and hope you will find this new version satisfactory for publication.

Please see our responses below, and do not hesitate to contact us if you would like further revisions made.

On behalf of all the authors,

Professor Lars E. Eriksson

Journal Requirements:

When submitting your revision, we need you to address these additional requirements.

1. Please ensure that your manuscript meets PLOS ONE's style requirements, including those for file naming. The PLOS ONE style templates can be found at

<https://journals.plos.org/plosone/s/file?id=wjVg/PLOSOne_formatting_sample_main_body.pdf> and 
<https://journals.plos.org/plosone/s/file?id=ba62/PLOSOne_formatting_sample_title_authors_affiliations.pdf>

Response: These style requirements have been consulted and adhered to.

2. Please review your reference list to ensure that it is complete and correct. If you have cited papers that have been retracted, please include the rationale for doing so in the manuscript text, or remove these references and replace them with relevant current references. Any changes to the reference list should be mentioned in the rebuttal letter that accompanies your revised manuscript. If you need to cite a retracted article, indicate the article’s retracted status in the References list and also include a citation and full reference for the retraction notice.

Response: Reference list reviewed. Four new references added as a result of the Reviewers’ comments.

**Reviewer: 1**

General
The authors should be consistent in writing “Covid-19”.It is sometimes mentioned starting in capital letter (e.g. line 38) and sometimes starting in small letter (e.g. line 33).

Response: Many thanks for a thorough review with constructive suggestions. We have changed to the WHO recommended format COVID-19 throughout the manuscript.

Introduction
Line 95: “qualitative studies confirm this” Reference of qualitative study should me mentioned in the same sentence (Reference number 16)

Response: Reference 16 added in the same sentence.

Cognitive interviews
Line 178-182
How did the authors select the participants for Think-aloud cognitive interviews?

Response: The participants were purposively selected (added in line 179) to represent different genders, ages and origins.

Analysis
Line 205
“Written records from the cognitive interviews were analysed by MR who summarised comments and 206 remarks item by item, which formed part of the validity assessment.”
It’s better to mention who performed the analysis in authors contribution section not within the manuscript

Response: We agree and have rewritten this sentence to not mention who performed this part of the analysis (lines 203-204).

Results
Table 1. Characteristics of study participants
Data presentation of age is better than “Year of birth”
Merging of the cells of the same variable (e.g. gender) is better in data presentation

Response: Thanks for these suggestions to make tables more clear and easy to read. Year of birth has been changed to years and horisontal cell borders of cells of the the same variable have been merged.

Line 255-257
“To a direct question about whether the items were relevant in general, some participants responded that the items could be relevant for people with covid-19 but were not relevant for them”. What do you mean by “them”? People who recovered from Covid-19?

Response: Thank you for pointing out this unclarity. We have clarified by changing the sentence to: “…*some participants responded that the items could be relevant for other people with COVID-19 but were nor relevant for* ***them****selves*” (lines 255-257). Further, we also discuss a possible interpretation “*all participants in the cognitive interviews stated that the questions could be relevant for other people with COVID-19, with a possible interpretation being that this refers to persons with ongoing infection*” (lines 340-342).

Table 2
Merging of the cells of the same variable (i.e.scales) is better in data presentation

Response: Cell borders of the same variable have been merged in all tables where applicable.

Discussion
Line 374 “… although people with HIV in some contexts had reduced access to ART and the health service during the pandemic …”
What does ART stand for?

Response: Thank you for observing the unintroduced abbreviation. We have now replaced the abbreviation with “*antiretroviral therapy”*.

The following manuscript may be helpful in discussion:
Validity and Reliability of a COVID-19 Stigma Scale Using Exploratory and Confirmatory Factor Analysis in a Sample of Egyptian Physicians: E16-COVID19-S. A Mostafa, NS Mostafa, N Ismail. International Journal of Environmental Research and Public Health 18 (10), 5451

Response: Many thanks for making us aware of this study. We have how slightly rewritten the background to include a recent Egyptian study on the attitudes among the general population (lines 96-97) and the stigma experiences among physicians (lines 104-106). Furthermore, we have included results from the physician study in the discussion about the psychometric results (lines 319-322).

**Reviewer #2:**

The study presents original research on the development of an instrument to measure COVID-19 related stigma. The cross-sectional assessments and respective analyses are performed to a high technical standard and are described in sufficient detail. The article is presented in an intelligible fashion and is written in standard English.

Response: Many thanks for the thorough review with constructive suggestions.

One major comment refers to the results that people who had experienced COVID-19 reported low levels of covid-19-related stigma. This is stated in the abstract and text and it is a finding of the study. However, it seems, as authors state in the Discussion, that it would be more plausible to expect people to experience stigma during their COVID-19 event and that this stigma might not persist after the resolution of the disease. As such, it would be important to state, in the abstract and elsewhere, that covid-19-related stigma was not measured during the symptomatic phase of the disease.

Response: Yes, we agree and have added to the abstract (lines 32-33): ”*Cognitive interviews and cross-sectional surveys* ***were made after the acute phase of the illness*** *using...”.* Furthermore, in the main text we have clarified on line 198 ”...*period after their COVID-19* ***event***...” In line 192-193 it is stated that ”*Within about two to three months after their diagnosis, the COVID-19 Stigma Scale was distributed to*...” and in the Discussion on lines 337-346, we discuss around these circumstances.

Two related questions are: 1) Did some participants answer the questionnaire during their COVID-19 event? And if yes, did stigma scores differ according to when it was measured?, 2) Do authors have information about symptomatic infection? If yes, could scores be compared between the two groups: symptomatic vs not?

Response: No, the participants did not answer the questionnaire during their COVID-19 event. We agree that experience of symptoms may well affect experience of stigma but even if we can assume that most of the participants had experienced symptoms from their COVID-19 event, we do not have any information about symptomatology. Hospitalisation can be an indication of severity of symptoms, though, and when comparing the group of participants that had been hospitalised and those not hospitalised because of their COVID-19 event, there were no statistically significant differences between the groups. In the original submission we chose to not include this result, but we have now added them in the methods (lines 225-226) and results (lines 297-298) and included a new paragraph discussing this: ”*Severity of symptoms could be suspected to influence affected peoples’ experiences from going through a COVID-19 event. In the present study, we did not have any details about symptomatology of the participants although hospitalisation status could be assumed to be a marker of presence of severe symptoms. When comparing the group of participants that had been hospitalised in relation to their COVID-19 event with those who had not, similar to the results recently presented by Avila et al [36], hospitalisation status did not show any differences in relation to experiences of COVID-19-related stigma”* (lines 354-360).

Page 3/line 36: “HIV who had experienced a concomitant covid-19.” Though the sentence is likely correct, it might be easier to read if “event” or “episode” was added after covid-19. Though COVID-19 is the official name of the disease (in upper case), the text “a concomitant covid-19” reads awkward (as it would read if it was another disease, such as pneumonia or flu). Other instances such as this one can be found throughout the text.

Response: We agree and have added deleted “*a concomitant*” and added “*event*” at lines 30, 35, 39, 132, 136, 226, 298, 307, 317, 323, 355, 358 and 412. Furthermore, we changed to the WHO recommended format “COVID-19” throughout the manuscript.

Page 8/line 142: “The study reported in this article included people in Sweden who had had covid-19.” It is not clear how this sentence is informative and how it relates to the context of COVID-19 in Sweden.

Response: Many thanks for pointing this out, this sentence how have been deleted.

Page 10/line 199: Did people living with HIV respond to both instruments consecutively? Which one was answered first? Because the items are the same (except for the words HIV/COVID-19), could this have influenced their responses and therefore the results?

Response: Many thanks, the participants were free to choose in what order to answer the two scales. Althogh a relevant aspect, since we did not keep any record on the choosen order of the participants responses, we cannot investigate this further. We have added the fact that ”*the participants were free to choose the order in which to answer the two scales*”, lines 198-199 in the Methods section.
